# Serosurvey of anti‐rubella and anti‐measles IgG antibodies in young females in Jahrom, southern west Iran in 2012: A review of literature of the serological profile in Iran

**DOI:** 10.1002/jgf2.677

**Published:** 2024-02-25

**Authors:** Najmeh Mojarad, Fatemeh Forouzani, Zahra Mohammadi, Reza Shahriarirad

**Affiliations:** ^1^ School of Medicine Jahrom University of Medical Sciences Jahrom Iran; ^2^ School of Medicine Yasuj University of Medical Sciences Yasuj Iran; ^3^ Student Research Committee Shiraz University of Medical Sciences Shiraz Iran; ^4^ Thoracic and Vascular Surgery Research Center Shiraz University of Medical Science Shiraz Iran

**Keywords:** antibody, immunization programs, measles, rubella, vaccination

## Abstract

**Background:**

Rubella and measles are two highly contagious viral childhood diseases. However, the high possibility of brutal effects of both microorganisms during pregnancy has kept them emerging as a major public health issue. The current study aimed to investigate the seroprevalence of rubella and measles among 15–25‐year‐old females in southwest Iran.

**Method:**

This descriptive study was performed in Jahrom, southern west Iran during 2012. Serum samples from female individuals with an age of 15–25 years visiting main laboratories in our study location were collected and evaluated by a commercial ELISA kit for anti‐rubella and measles IgG antibodies.

**Results:**

Among the 179 participants, regarding anti‐rubella antibodies, 171 (95.0%) were positive, 3 (1.7%) were negative, and 6 (3.3%) were borderline. Regarding anti‐measles antibodies, 166 (92.2%) of the subjects were positive, 1 (5.6%) was negative, and 13 (17.2%) were borderline. By assuming a positive value for the borderline cases, the ultimate findings demonstrated that 98.3% and 99.4% of the participants were immune against rubella and measles, respectively. There was no statistically significant association between measles and rubella immunity with age or the use of immunosuppressor medication.

**Conclusion:**

Implementation of vaccine program has obtained significant immunity level in inhabitants, particularly women of childbearing age who play a more important role in herd immunity. Therefore, maintaining the national immune screening against rubella and measles is needed to take into consideration to maintain the current satisfactory level of immunization.

## 1 INTRODUCTION

Although measles and rubella are generally considered to be benign viral infections with mild symptoms in childhood, they are among notable viral diseases with devastating consequences on pregnancy outcome.^1^ Measles is a single‐stranded highly contagious RNA virus of the genus Morbillivirus and the family Paramyxoviridae which could cause systemic disease in previously unexposed person.^2^ More importantly, its exposure in the latest stages of pregnancy associated with higher risk of morbidity and mortality for mother and her fetus as well. Major threat for pregnant women is respiratory distress, which in worst case might cause death, also its transmission through placental may exert burdens on fetus including congenital infections, miscarriage, and prematurity. However, congenital anomalies have not been reported.^3^ Rubella virus, also known as German measles, is a member of the Rubivirus in the Matonaviridae family. If a pregnant woman acquires rubella virus in the first trimester of pregnancy, transmission to the fetus will reach to 90%.^1^ This can cause miscarriage, fetal death, stillbirth, or congenital anomalies (e.g., hearing loss, eye impairments, congenital heart disease, mental retardation, and neurological complications), which collectively have been known as congenital rubella syndrome (CRS).^4^, ^5^ To a large extent, both of these microorganisms present with the same manifestations in adults except for the pathognomonic exanthem (Koplik spots) occurring in measles disease, and a slight difference in the characteristic of their maculopapular rashes.^6^

With respect to these dramatic influences on both mother and fetus, the main aim of vaccination program is to mitigate the risk of infection during pregnancy. Rubella vaccination has been implemented in many countries in the mid‐1960s and later via vaccines that also contain live attenuated measles and mumps viruses.^7^ Iran typically has a vaccination rate of over 95%.^8^ Epidemiological studies have shown that vaccination rates probably do not accurately reflect the community's degree of immunity. There have been few studies on whether teenagers and childbearing women who received the MMR vaccine a few years ago have antibodies against it. The bulk of the studies that have been highlighted recently used small sample sizes, and the majority of them are outdated.^9^, ^10^, ^11^, ^12^, ^13^, ^14^ As a result, the immunity situation for teenagers who received vaccinations is unclear, and the data currently available are insufficient to guide policy.

In Iran, vaccination against measles was launched in 1983 for the first time and to ensure continued high population protection, a routine second dose was scheduled in 1985. According to the National Immunization Program of Iran, all Iranian children received MMR1 (measles, mumps, and rubella) vaccine at 12 months and then at 4–6 years of age for MMR2 free of charge. This policy was changed in 2007, and children are vaccinated in 18 months for the second dose.^15^, ^16^

In the nationwide vaccination campaign of December 2003 of Iran, all the population ranging from 5 to 25 years participated to receive shots against rubella and measles (roughly 33 million people), afterward the regional campaign of November 2015 was the largest one.^17^ In comparison to the other Middle Eastern countries, vaccination coverage against measles and rubella viruses in the past two decades in Iran has been become much higher with a remarkable rise from 72% in 1984 and 78% in 1987 to around 99% in 2011.^18^ In 2019, WHO announced that measles and rubella have been successfully eliminated in Iran as the third country of Middle East after Bahrain and Oman with the population of 80 million people.^19^ In a study by Saffar et al. in Iran, 12.3% and 18.4% of the population lacked antibodies to rubella and measles, respectively. After revaccination, 92 and 94.9%, respectively, of seronegative people demonstrated IgG response to the measles and rubella vaccines. However, a significant portion of vaccine recipients were seronegative for measles and rubella despite the high coverage rate of measles and rubella vaccinations, which may be due to secondary vaccine failure and may have a negative impact on the nation's goals for measles‐rubella eradication. Therefore, more substantial regional or national supplementary immunization program has to be taken into consideration if these findings are supported by similar future studies.^20^

The prime intention of current study was to monitor the impact of the public vaccination program against rubella and measles in women of childbearing ages in order to find out whether is essential to impose a new healthcare policy for extra inoculation in this target group or simply sustain the former program. We evaluated the efficacy of our vaccination program based on the seroprevalence of measles and rubella in our study location, which has been widely used in literature.^1^, ^21^, ^22^ Also, we reviewed the results of other studies conducted in different parts of Iran regarding the seroprevalence of rubella and measles to reach a better understanding about the level of immunity in the whole country.

## 2 METHOD

### 2.1 Study design and data collection

In this descriptive study, female individuals aged 15–25 years were included. Based on the community‐oriented nature of our study, the sample size was estimated as 179 patients. Available census sampling method was considered for this study, in which 179 female individuals between the age of 15–25 years who were visiting referral certified university‐affiliated laboratories in Jahrom, southern west Iran during 2012 were included in our study. The aim of the study was described to the patients and their voluntarily involvement was requested. The sample size consisted of all female participants visiting these centers for any reason (including routine, pre‐pregnancy, or pre‐marriage screening test, or referred from clinics or hospitals for laboratory evaluation) within the age limit and willing to participant in the study. The patients' age, along with the history of immunosuppressor medication, including corticosteroids or any other immunosupperesive medications used for autoimmune disorders, use was collected.

### 2.2 Study location

Our study was conducted in Jahrom, the second largest city in Fars Province, southern west Iran. Agriculture has always been a major part of the economy in Jahrom. The main ethnic groups in Jahrom consists of Persians, while Arabs and Basseries constitute small minorities. Based on the geographical characteristics of Jahrom, especially good Rangeland for nomads, Jahrom has long been a residing area for these nomadic peoples.

The study was approved by the Ethics committee of the University (ethical code: IR.TUMS.IKHC.REC.1399.075). The purpose of the study was explained to each participant by the researcher, and after obtaining written informed consent, clot blood samples were collected. The samples were centrifuged at 4°C and stored in a freezer. After obtaining the required number of samples, anti‐rubella and anti‐measles IgG antibodies were evaluated by ELISA method.

### 2.3 Detection of anti‐rubella and anti‐measles antibodies

ELISA kits (Vircell Spain SLU, Granada, Spain) were used similar to previous studies,^23^, ^24^ with a preliminary sensitivity and specificity of 97% and 94% for the detection of anti‐measles IgG and 95.5% and 97.1% for anti‐rubella IgG. All procedures were performed in accordance with the kit manufacturer's guidelines. The samples were placed at room temperature for 1 h, and 5 μL serum was drawn and subsequently diluted with 100 μL of dilution serum. Also, 5 μL serum samples were placed for positive and negative controls. The sera were mixed for 2 min via shaker and placed in an incubator at 37°C for 45 min. The samples were then washed five times with 0.3 μL washing buffer, and then 100 μL of IgG was added to all samples, and subsequently placed in the incubator at 37°C for another 30 min. Afterward, plates were washed as before, and 100 μL of substrate was added. After 20 min of incubation at room temperature and in dark, 50 μL of stopping solution was added. The OD at 450–620 nm was measured, using an ELISA plate reader.

Based on the kit instructions, samples in which their anti‐measles and anti‐rubella IgG were lower than 9 IU/mL were assigned as negative, between 9 and 11 as borderline, and above 11 as positive. We also rounded up our results and merged borderline cases with the positive cases. The method of detection was similar to previous studies and the commercial kit guidelines.^23^, ^25^, ^26^, ^27^, ^28^

### 2.4 Statistical analysis

All obtained data were entered into SPSS version 22.0 and reported in a descriptive manner with frequency and percentage or mean and standard deviation (SD). The chi‐square test was used to evaluate the association between variables. A *p*‐value of less than 0.05 was considered statistically significant.

## 3 RESULTS

In our study, 179 female individuals between 15 and 25 years were evaluated for anti‐measles and anti‐rubella IgG antibodies. Based on our results, regarding anti‐rubella antibodies, 171 (95.0%) were positive, 3 (1.7%) were negative, and 6 (3.3%) were borderline. Regarding anti‐measles antibodies, 166 (92.2%) of the subjects were positive, 1 (5.6%) was negative, and 13 (7.2%) were borderline. By assuming a positive value for the borderline cases, the ultimate findings demonstrated that 98.3% and 99.4% of the participants were immune against rubella and measles, respectively (Table 1).

**TABLE 1:** Evaluation of rubella and measles IgG antibodies based on age and the use of immunosuppressor and corticosteroid medication.

Variable		Total; *N* = 179	Rubella IgG, (%)			*p*‐value	Measles IgG, (%)			*p*‐Value
			Immune; *N* = 170	Non‐immune; *N* = 3	Borderline; *N* = 6		Immune; *N* = 165	Non‐immune; *N* = 1	Borderline; *N* = 13	
Immunosuppressive Medication	Yes	6 (13.4)	6 (100)	0 (0)	0 (0)	0.848	5 (83.3)	0 (0)	1 (16.7)	0.656
	No	173 (96.6)	164 (94.8)	3 (1.7)	6 (3.5)		160 (92.5)	1 (0.6)	12 (6.9)	
Age	15–19	41 (23.2)	40 (97.6)	0 (0)	1 (2.4)	0.581	38 (92.7)	1 (2.4)	2 (4.9)	0.486
	20–25	136 (76.8)	128 (94.1)	3 (2.2)	5 (3.7)		124 (91.2)	1 (0.7)	12 (8.8)	

There was no statistically significant difference among the age groups regarding rubella and measles immunity (*p* > 0.05). Also, there was no significant association between immunosuppressive medication use and seropositivity to rubella or measles (*p* > 0.05).

## 4 DISCUSSION

The foremost purpose of the current study was to monitor the impact of a public vaccination program against rubella and measles by the immune status of women of childbearing ages after more than 9 years of nationwide MMR vaccination campaign of December 2003. Besides the herd protection factor, preventing teratogenic effects and fortifying fetus immune system with placenta antibodies all led to the exclusive assessment on reducing rubella and measles by immunizing this particular group. In the nationwide vaccination campaign of December 2003 of Iran, all the population ranging from 5 to 25 years participated to receive shots against rubella and measles (roughly 33 million people), afterward the campaign of November 2015 was the largest one.^17^ Several studies were conducted to evaluate the efficacy of these mass campaigns.

The total findings of the present study confined a significant and satisfactory level of sero‐immunity against measles and then against rubella reporting as 99.4% and 98.3% (antibody value > 9 IU/mL).

This immunity rate in our study is comparable to those were reported in previous studies at the national level. Other surveys in various provinces of Iran showed that the elimination vaccine program of rubella has accelerated the seropositivity among women at the verge of marriage, hence the index in Qom, Mashhad, Shahre‐Kord, Hamadan, Sari, Tehran, and Kermanshah in 2012 were 98.4%, 89.5%, 96.9%, 98.4%, 93%, 97.4%, and 99.3%, respectively, and 98.9% in Shiraz in 2004, suggesting efficacy of vaccination in providing sufficient immunity in target groups.^21^, ^29^ Another survey showed that 1 year after the vaccination campaign of 2003, rubella immunity reached 91.0%, 99.6%, 99.6%, and 97.0%, respectively, for the 6–10, 11–15, 16–20, and 20–26 years old age groups. Seropositivity for the rubella virus in the target population was high, especially in women of childbearing age (98.9%), close to the 98.3% immunity level of our study. However, this study was not related to rubella vaccination in 2003 of the first group.^21^

Considering the seroprevalence of measles in a different part of the country, a study in Hamadan province in 2018 showed 63.24% population of 1–40 years old had positive IgG against measles.^30^ To some extent, the difference in the characteristic of studies population (such as age, sex, and vaccination history) can explain the low percentage of immunity.

Additionally, an earlier study reported that seropositive prevalence against measles based on different age groups reached 80.6% (6–10 years group), 72.7% (11–15 years group), 84.9% (16–20 years group), and 87.5% (20‐26 years group). Although, there is no data about the seroprevalence of different sex groups of mentioned study the seropositivity was lower compared with the current one.^21^

In a nationwide study in 2015, 1231 (65.8%) positive tests for measles, and 1344 (80.1%) for rubella were detected among a population of children and adolescents in Iran.^15^ Based on these findings, the authors stated that IgG antibodies against measles and rubella were not found in a sizable part of kids and teenagers aged 7 to 18 in Iran, despite the country's high immunization rates. Iranian children may experience issues if they come into touch with people from other nations where the measles or rubella have not yet been eradicated. Later seroprevalence studies in 2023 on anti‐measles immunoglobulin G antibody showed that a booster vaccination against measles may be necessary due to the inadequate levels of immunization.^31^ Hence, although mass campaign demonstrated sufficient efficacy at the time of our study and Regional Verification Commission for Measles and Rubella Elimination has declared that measles and rubella have been eliminated in Iran, some notable challenges have reduced this outcome in recent years.^32^

The level of rubella IgG antibodies in females in the current survey (98.3% immunity) was higher than most studies, demonstrating effective immunity following vaccination programs in our country. In several studies conducted in Korea between 2010 and 2017, rubella seronegativity among women aged 15–49 years was between 7.8% and 9.7%.^33^ The percentage of pregnant women who were immune to rubella in 2017 was 87.8% in India and 95.4% in Brazil.^34^, ^35^ Protective levels of anti‐rubella IgG were documented in pregnant women, and Rubella seroprevalence among women of childbearing age was reported as 87% in Kyrgyzstan (2001),^36^ 86.1% in Turkey (2015),^37^ 83.9% before (2000–2001), and 93.5 after (2002–2003) the campaign in Brazil,^22^ 58.4% in China (2010–2012),^38^ 88.2% in Egypt (2010),^39^ and 84.6% in Hongkong (1997).^40^ The immunity level against measles in a vast survey including 18 European countries between 1996 and 2004 significantly varied; Thus, findings revealed seven countries (Czech Republic, Hungary, Luxembourg, Spain, Slovakia, Slovenia, and Sweden) met extensively close to the WHO elimination targets (<15% in those aged 2–4 years, <10% in those aged 5–9 years and <5% in those aged 10–19, 20–39, and 40+ years.). Four countries (Australia, Israel, Lithuania, and Malta) had susceptibility levels (targets missed in two of the following age groups: 10–19, 20–39 or 40+) in some older age groups suggesting possible gaps in protection. Seven countries (Belgium, Bulgaria, Cyprus, England, and Wales, Ireland, Latvia, and Romania) were regarded to be at risk of epidemics due to the high susceptibility in children and also adults (targets missed in either 2–4 or 5–9‐year‐old age groups).^41^ Although the previous paper was widely different compared with the present one deeming the features of the target group, especially age and sex.

Other studies conducted in Italy (2016–2017) and France (2009–2010) showed that approximately 96.9% and 80.59% of pregnant women were positive for anti‐measles IgG respectively and the first study was very close to our results.^42^, ^43^

The noticeable difference in anti‐rubella and anti‐measles antibodies in different nations can be justified by the routine vaccination program, implementation of any mass campaign, level of public sensitization, and awareness. In a previous study in 2017 in Iran by Zahraei et al.,^19^ anti‐measles and anti‐rubella antibodies were present in overall seroprevalences of 90.6% (95% CI: 89.1 to 92.0%) and 80.7% (95% CI: 78.7 to 82.6%), respectively. They concluded that the 2003 measles and rubella vaccination campaign still had an impact on the age group of 5 to 25 years after 14 years, as evidenced by the stark difference in seroprevalence of antibody (against both measles and rubella) between those who were over 18 at the time of the present study and the younger age cohorts. Women older than 18 were found to have greater seroprevalences of antibodies for both illnesses. The authors stated that to close the gap in the sero‐immunity of the younger age groups, it may be necessary to implement a Supplemental Immunization activity or revise the national immunization schedule to include a third dose of the measles and rubella‐containing vaccine during adolescence.^19^

When looking more meticulously at Table 1 and considering the range ages of 15–19 and 20–25 the positive immunity reached 97.5% and 94.2% against rubella, 92.6%, and 90.5% against measles, respectively; however, there was no statistically significant difference among the groups. Although earlier studies in 2004 and 2011 have suggested a decrease in serum IG titers by aging,^1^, ^21^ our study demonstrated that in these age groups, immunity rates against these two diseases were parallel; however, further studies in larger populations and higher aged females are justified to evaluate the exact extent of the impact of age on vaccination immunity.

Furthermore, we aimed to evaluate the contribution between immunosuppressive medication use and low concentration of anti‐rubella and anti‐measles antibodies. Out of six precipitants with a positive history of mentioned factors, none of them were seronegative for rubella and only 1 was assumed to be borderline against measles. Hence, there was no remarkable association between these factors. A study in 2015 in line with this finding claimed all patients with inflammatory bowel disease had measurable antibody concentrations to the three vaccine viruses (MMR). Also, they had detected no difference in the antibody concentration of rubella, measles, and mumps among the inflammatory bowel disease group and health control.^44^

### 4.1 Limitations

It was impossible to compare the immunity level in different categories with focusing on socio‐economic status because of the random sampling method. However, the major issue was a rapid rise in kits expenses causing an obligation in decreasing the sample size to 179 substitutes 200 sera. Besides, there was a several month's time gap between sampling and data analysis which may lead to borderline results of few samples. Based on the kit's instructions and expert's experience, these results are considered as positive findings in final data inevitably. Other limitations include the population of our study and small sample size, which was only among participants visiting our referral laboratories, and may not be a total representative of the general population and all childbearing females. We also considered borderline cases as positive cases, to narrow the margin of error and round up the number of seropositive cases based on the clinical significance and the desire to minimize the risk of false negatives. Further multicentral larger population studies, while including both male and female participants, are required to have an overall estimation of the current status and immunity of rubella and measles in the community.

## 5 CONCLUSION

Overall, we demonstrated high rates of immunity to rubella and measles after 9 years following the elimination program. On one hand, we believe that these results may support the effectiveness of the community vaccination program in order to alleviate the burdensome impacts on pregnancy outcome. On the other hand, recent surveys have shown a significant decline in public immunization and potential challenges may pose a risk to the current status. We suggest that further nationwide surveys with larger population sizes will be needed to determine the need for national supplementary immunization activities or anti‐rubella and anti‐measles screening among women of childbearing age.

## AUTHOR CONTRIBUTIONS

N.M. and R.S. designed the study. F.F. collected the data. R.S. and Z.M. drafted the manuscript. All authors read and approved the final version of the manuscript.

## CONFLICT OF INTEREST STATEMENT

The authors have stated explicitly that there are no conflicts of interest in connection with thisarticle.

## ETHICS APPROVAL STATEMENT

The present study was approved by the Medical Ethics Committee of Jahrom University of Medical Sciences (ethical code: IR.TUMS.IKHC.REC.1399.075). The purpose of this study was completely explained to the participants and were assured that their information will be kept confidential by the researchers.

## PATIENT CONSENT STATEMENT

Written informed consent was obtained from the participants for the publication of this report and any accompanying images. A copy of the written consent is available for review by the Editor of this journal.

## Data Availability

All data regarding this report has been reported in the manuscript. Please contact the corresponding author in case of requiring any further information.
